# Individualized survival prediction and surgery recommendation for patients with glioblastoma

**DOI:** 10.3389/fmed.2024.1330907

**Published:** 2024-05-09

**Authors:** Enzhao Zhu, Jiayi Wang, Qi Jing, Weizhong Shi, Ziqin Xu, Pu Ai, Zhihao Chen, Zhihao Dai, Dan Shan, Zisheng Ai

**Affiliations:** ^1^School of Medicine, Tongji University, Shanghai, China; ^2^Department of Anesthesiology and Perioperative Medicine, Shanghai Fourth People's Hospital, School of Medicine, Tongji University, Shanghai, China; ^3^Shanghai Hospital Development Center, Shanghai, China; ^4^Department of Industrial Engineering and Operations Research, Columbia University, New York, NY, United States; ^5^School of Business, East China University of Science and Technology, Shanghai, China; ^6^School of Medicine, Royal College of Surgeons in Ireland, University of Medicine and Health Sciences, Dublin, Ireland; ^7^Faculty of Health and Medicine, Lancaster University, Lancaster, United Kingdom; ^8^Department of Medical Statistics, School of Medicine, Tongji University, Shanghai, China; ^9^Shanghai Pudong New Area Mental Health Center, School of Medicine, Tongji University, Shanghai, China

**Keywords:** glioblastoma, neurosurgery, deep learning, treatment recommendation, causal inference

## Abstract

**Background:**

There is a lack of individualized evidence on surgical choices for glioblastoma (GBM) patients.

**Aim:**

This study aimed to make individualized treatment recommendations for patients with GBM and to determine the importance of demographic and tumor characteristic variables in the selection of extent of resection.

**Methods:**

We proposed Balanced Decision Ensembles (BDE) to make survival predictions and individualized treatment recommendations. We developed several DL models to counterfactually predict the individual treatment effect (ITE) of patients with GBM. We divided the patients into the recommended (Rec.) and anti-recommended groups based on whether their actual treatment was consistent with the model recommendation.

**Results:**

The BDE achieved the best recommendation effects (difference in restricted mean survival time (dRMST): 5.90; 95% confidence interval (CI), 4.40–7.39; hazard ratio (HR): 0.71; 95% CI, 0.65–0.77), followed by BITES and DeepSurv. Inverse probability treatment weighting (IPTW)-adjusted HR, IPTW-adjusted OR, natural direct effect, and control direct effect demonstrated better survival outcomes of the Rec. group.

**Conclusion:**

The ITE calculation method is crucial, as it may result in better or worse recommendations. Furthermore, the significant protective effects of machine recommendations on survival time and mortality indicate the superiority of the model for application in patients with GBM. Overall, the model identifies patients with tumors located in the right and left frontal and middle temporal lobes, as well as those with larger tumor sizes, as optimal candidates for SpTR.

## Introduction

Glioblastoma (GBM) is an aggressive and invasive malignant neoplasm, which is the most common type of malignant brain tumor in adults (1), with a 5-year survival rate of only 5% (2) and a median overall survival (OS) time of approximately 15 months (3). The poor prognosis of GBM highlights the importance of identifying significant variables that can predict survival time in patients diagnosed with GBM. Although previous studies have demonstrated age, sex, extent of resection (EOR), preoperative magnetic resonance imaging (MRI) characteristics of tumors, degree of necrosis, and Karnofsky Performance Status Scale score as prognostic factors (4, 5), the results of these studies are mainly obtained from a group of participants. The lack of individualized consideration limits the practical guidance of these variables for treatment selection and survival prediction.

The EOR is one of the strongest prognostic factors that may contribute significantly to extended survival time. It can range from biopsy to subtotal resection (STR), gross total resection (GTR), and supratotal resection (SpTR). The optimal EOR considering all demographic factors and tumor features, risks, and benefits of resection to extend patient survival remains controversial. Although most of the previous studies have highlighted the significance of receiving a maximal EOR (6), the delicate structure of the brain and the risk of injuring nerves and blood vessels, especially owing to the widespread and diffusely infiltrating characteristics of GBM, make this goal difficult to attain (1).

Among the aforementioned treatment options, the superior selection between GTR and SpTR remains uncertain. GTR leads to lower disease progression and higher survival compared with STR. However, even with GTR, tumor recurrence at or near the primary resection site is inevitable (7). SpTR was defined as the EOR of GTR with some non-contrast-enhanced resection added to it, and studies in GBM have demonstrated that, compared to GTR, SpTR was associated with longer OS without new postoperative deficits (8). Therefore, in recent years, several studies have focused on the use of SpTR in GBM (3, 9), but the insufficient number and quality of relevant studies and the heterogeneity between the results of different studies have made its use highly controversial. Therefore, the treatment recommendation section of this study focused on GTR and SpTR.

Owing to the expensive implementation costs and ethical constraints of randomized controlled trials (RCTs), the analysis of causal effects directly through observational studies is efficient and inexpensive. Furthermore, we aimed to clarify how an individual patient or a specific group of patients will respond to the intervention. However, the finding of average treatment effect (ATE) does not necessarily hold at the individual level. The individual treatment effect (ITE) can only be obtained by inferring from data (10). With the ideal way of including treatment as a covariate (11), although it is predictive, as the model will be biased from confounders if the treatment is not allocated randomly (12), it is not an unbiased estimate. Alternatives include conditional average treatment effect (CATE)- (13), matching- (14), and representation-based approaches (15).

Regarding semi-parametric time-to-event survival regression, which is the most popular survival analysis tool (16), the calculation of the outcome of interest varies (17, 18) because the time-to-event outcome is a time tendency rather than a single point. However, surprisingly, few researchers using machine learning (ML)-based treatment recommendations have studied the effects of different ITE calculation methods, considering their significant role in treatment evaluation and clinical interpretability.

This study aimed to determine the importance of demographic and tumor characteristic variables in the selection of EOR and to provide a focus and basis for clinicians when making treatment decisions. Furthermore, in this study, we compared two methodologies for calculating ITE and combined them with Balanced Individual Treatment Effect for Survival data (BITES) (19), which is one of the latest deep learning (DL)-based survival regression models, to make better surgical recommendations for patients with GBM.

## Methods

### Study design

This was a retrospective cohort study predicting the survival outcomes of patients with GBM and identifying the patients’ ITE to determine whether an individual is better suited to receive GTR or SpTR with DL models. All participants included in this study were selected from the Surveillance, Epidemiology, and End Results 18 (SEER 18) database, which tracks patients with cancer from 18 regions of the United States, and the population in SEER 18 represents approximately 27.8% of the US population (20). This study followed the Strengthening the Reporting of Observational Studies in Epidemiology (STROBE) reporting guidelines (21).

**Figure 1:**
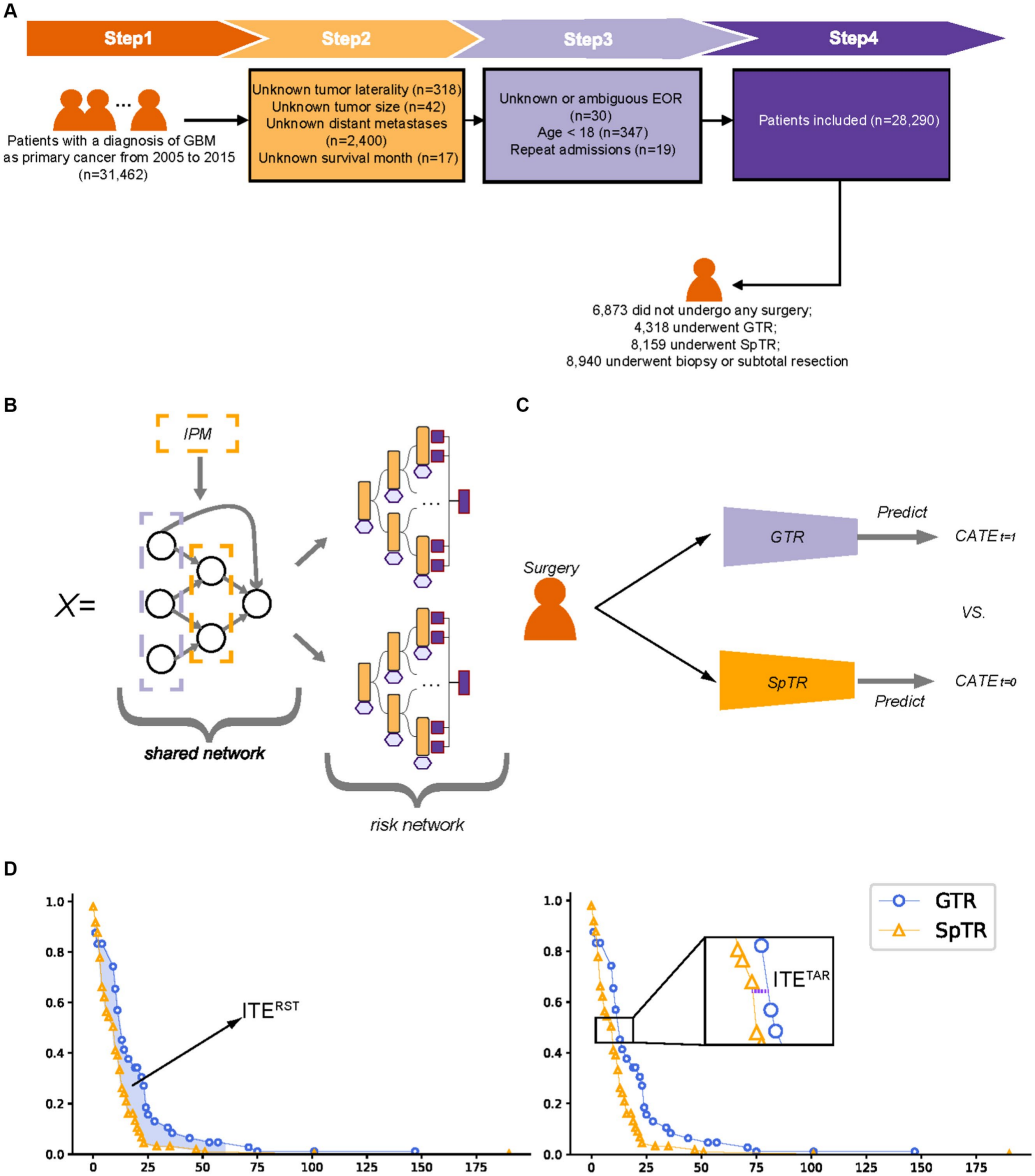
Patient inclusion flowchart, model structure schematic, and individual treatment effect calculation schematic. **(A)** Patient inclusion flowchart; **(B)** balanced Decision Ensembles structure schematic; **(C)** T-learner structure schematic; **(D)** The calculation of the individual treatment effect. GTR, gross total resection; SpTR, supratotal resection; CATE, conditional average treatment effect; ITE, individual treatment effect; RST, restricted survival time; TaR, time at risk.

Patients diagnosed with GBM as a primary cancer from 2005 to 2015 were included in this study. The exclusion criteria were as follows: (1) age less than 18 years; (2) unknown tumor location, laterality, or size; (3) unknown or ambiguous EOR; (4) unknown survival time; and (5) repeated admissions. The overall study population inclusion process is illustrated in Figure 1A. We collected baseline patient information (sex, age, marital status, living area, economic status, and reporting state), tumor-related information (tumor size, primary location, laterality, extension, and metastasis), and treatment details (surgical types). The tumor size, referring to the tumor diameter, was recorded at the time of GBM diagnosis. The outcome of interest was brain cancer-specific survival (BCSS) provided by the SEER, which indicates the time interval between death caused by a brain tumor and diagnosis of GBM.

### Deep learning architecture

BITES contains a shared network, a multilayer perceptron (MLP), and two risk networks, two MLPs, and each risk network represents a specific treatment. BITES calculates the losses of two treatments separately and combines them with integral probability metrics (IPM) regularization, a causality estimation based on representation learning (22), to balance the generating distributions of different treatment groups. Treatment-specific baseline hazards were calculated before the inference.

We performed a simple but effective modification of BITES, called Balanced Decision Ensembles (BDE), to enhance the ability of feature extraction and to speed up inference. We used LassoNet (23) to replace the shared MLP and two Neural Oblivious Decision Ensembles (NODE) (24) to replace the risk MLPs. The architecture of LassoNet consists of a single residual connection, a linear component, and a non-linear component. LassoNet allows a feature to participate in the non-linear part only if its penalized linear representation is active. Therefore, it reduces the influence of irrelevant features and has lower computational cost and better generalizability. NODE uses oblivious decision trees (ODTs) as weaker learners and inherits the classic hierarchical DL architecture. An ODT places a constraint on a regular decision tree that uses the same splitting feature and threshold in all internal nodes of the same depth. ODTs are not easily overfitted and are computationally efficient (25). NODE prediction is obtained by weighting the ODTs of each layer. The overall structure of BDE is presented in Figure 1B.

For DeepSurv, a treatment recommendation system was developed by separately training models on the GTR and SpTR training sets, which can be called T-learner (13). The individual survival curves predicted by these two models were then compared for the different treatments (Figure 1C). In this study, the recommendation of the Cox proportional hazards (CPH) model and random survival forest (RSF) was obtained in the same way as T-learners.

In treatment recommendation tasks, these models predict potential log hazard ratios based on patients’ baseline preoperative characteristics under the hypothesis of different treatments (GTR and SpTR), respectively. The log hazard ratios and treatment-specific baseline hazards are transformed by the Kaplan–Meier (K–M) method to obtain the individual survival distribution of patients, presented as the curve of survival probability of individual patients over time during the follow-up period. Based on this survival distribution, the ITE can be calculated and the treatment plan with comparative survival advantages can then be obtained, termed as treatment recommendation. When making survival predictions, models predict patients’ log hazard ratios regardless of the surgical type. The baseline hazard was calculated based on their actual survival in the training set. The individual survival distribution was obtained in the same way as mentioned above.

### Individual treatment effect

The ITE calculation process is illustrated in Figure 1D. In estimating ITE, only a single factual can be observed per patient, whereas the outcome of the alternative situation is missing. Hence, for simplicity, ITE can be defined as $\mathrm{IT}E_{i}={Y_{i}}^{T=1}\left(X_{i}\right)-{Y_{i}}^{T=0}\left(X_{i}\right)$ , where $Y_{i}$ is the outcome of a situation of patient i , which can be measured in different ways, T indicates different surgical interventions, and $X_{i}$ is the covariate. A patient either received a treatment of T=0 or T=1 , whereas the other situation was called counterfactual. Fortunately, counterfactual survival outcomes can be predicted using ML models.

In this study, we used two methods with good clinical interpretation to calculate the outcomes ( $Y_{i}$ ) in the ITE calculus: the time at risk (TaR) and restricted survival time (RST). The former was defined as the time for an individual to reach a specific mortality rate, which was close to the definition of median survival time (MST), as we took the time when the mortality rate was 50%. The latter was defined as the area under the survival curve of an individual in a specific time period (5 years), which was close to the definition of restricted mean survival time (RMST), which described the mean survival time of the subject population during the follow-up period. An ITE with higher values indicates a better survival outcome (e.g., an ITE greater than zero indicates patients are likely to achieve better BCSS with SpTR compared to GTR) and, thus, will be recommended by the model.

### Model development and treatment recommendation

All patients were randomly allocated to a training set of 80% of the samples that were used for building the models and a testing set of 20% of the samples to evaluate the model performance and the effect of the models’ recommendation. During the training period, we used fivefold cross-validation to tune the model hyperparameters; for each time, the model was trained on four-fifths of the training set and validated on the remaining one-fifth of the training set. The training process was terminated automatically if the validation loss did not decrease in 1,000 iterations.

To explore the effects of the recommendations, we divided the patients into the recommended (Rec.) and anti-recommended (Anti-rec.) groups, based on whether the actual treatment they received was consistent with the model recommendations. Except for the concordance index (C-index) and integrated Brier score (IBS), we calculated the difference in RMST (dRMST) and hazard ratio (HR) as two core metrics to evaluate recommendation effectiveness, as they quantified and directly responded to better survival outcomes in the Rec. group than in the Anti-rec. Group. These indicators have sufficient clinical interpretability and statistical guarantees.

### Model interpretation and visualization

SHapley Adaptive exPlanations (SHAP) is a widespread model-agnostic local explanation based on the Shapley value framework of game theory. Shapley values explain the extent to which each variable affects the model output relative to the baseline average. We used SurvSHAP(t) (26), which is capable of providing model explanations in the form of survival function rather than a single point or aggregation (27), to make time-dependent explanations for our models.

Additionally, we developed a user-friendly interface to facilitate survival predictions and treatment recommendations from the model with the best recommendation effectiveness. A user can input a comma-separated value (CSV) file that contains the required features. The survival probability, regardless of treatment, will be predicted by clicking the “predict” button. Treatment recommendations can be obtained by clicking the “recommend” button, followed by two types of ITE based on specific individual information. Once a CSV file of multiple patients is uploaded, the user can switch to the next patient by choosing the patient ID.

### Statistical analyses

Statistical analyses were performed using R 4.1.3 and Python 3.8. Continuous variables are reported as medians and interquartile ranges (IQRs), and categorical variables are presented as numbers and percentages (%). The log-rank test was used to compare K–M curves. We established a logistic regression to predict model recommendations from covariates to explain the behavior of the model recommendation.

## Results

### Demographic status and clinicopathology

Based on the inclusion and exclusion criteria, 28,290 patients with BCSS records were included in this study. The baseline clinical characteristics of all patients, those who underwent GTR, and those who underwent SpTR are presented in Table 1. Regarding surgery information, 6,873 (24.3%) patients did not undergo any surgery, 4,947 (17.5%) underwent biopsy, 3,993 (14.1%) underwent STR, 4,318 (15.3%) underwent GTR, and 8,159 (28.8%) underwent SpTR. The median (IQR) age was 64 (55–73) years; 58.1% were men; the majority of patients were white (89.8%) and were from urban areas (87.9%) and the states of the midwestern United States (64.6%); and 71% of the patients had household income of more than $55,000, which was the estimated median annual US household income in 2015 (28). The overall incidence rate of BCSS was 83.4% (95% confidence interval [CI], 83.0–83.9%) over a median (IQR) follow-up time of 8 (3–18) months. Among the tumor-related variables, the sites with the highest incidence of tumors in the total population were the frontal (7,981 [28.2%]), temporal (7,044 [24.9%]), and parietal lobes (4,583 [16.2%]) and overlapping (tumors that involved two or more lobes) regions (6,024 [21.3%]). Most of the tumors were lateralized to the left (11,538 [40.8%]) and right (12,123 [42.9%]) sides, and fewer were located in the middle (4,629 [16.4%]). In 21,523 (76.1%) patients with GBM, the tumors were confined *in situ* without extension, and only 4,493 (15.9%) crossed the midline. Only 398 (1.4%) had metastases. The distribution characteristics of the above tumor-related variables in patients undergoing SpTR and GTR were similar to those of the total population.

**Table 1:** Demographic status and clinicopathology.

	Overall (*n* = 28,290)	GTR (*n* = 4,318)	SpTR (*n* = 8,159)
Age, median (range), y	64 (55–73)	63 (54–70)	62 (53–71)
Tumor size, median (range), mm	45 (33–56)	45 (33–56)	45 (33–56)
Sex			
Female	11,852 (41.9%)	1819 (42.1%)	3,316 (40.6%)
Male	16,438 (58.1%)	2,499 (57.9%)	4,843 (59.4%)
Race			
White	25,394 (89.8%)	3,900 (90.3%)	7,378 (90.4%)
Others	2,896 (10.2%)	418 (9.7%)	781 (9.6%)
Married			
Yes	18,050 (63.8%)	2,867 (66.4%)	5,466 (67.0%)
No	10,240 (36.2%)	1,451 (33.6%)	2,693 (33.0%)
Urban			
Yes	24,873 (87.9%)	3,849 (89.1%)	7,204 (88.3%)
No	3,417 (12.1%)	469 (10.9%)	955 (11.7%)
Area of the United States			
Midwest	18,276 (64.6%)	2,867 (66.4%)	5,263 (64.5%)
East	4,720 (16.7%)	672 (15.6%)	1,583 (19.4%)
South	5,008 (17.7%)	755 (17.5%)	1,250 (15.3%)
Oversea	286 (1.0%)	24 (0.6%)	63 (0.8%)
Income (US dollar)			
Lower than $55,000	8,198 (29.0%)	1,325 (30.7%)	2026 (24.8%)
Higher than $55,000	20,092 (71.0%)	2,993 (69.3%)	6,133 (75.2%)
Location			
Frontal	7,981 (28.2%)	1,305 (30.2%)	2,351 (28.8%)
Temporal	7,044 (24.9%)	1,302 (30.2%)	2,440 (29.9%)
Parietal	4,583 (16.2%)	786 (18.2%)	1,374 (16.8%)
Occipital	1,223 (4.3%)	236 (5.5%)	379 (4.6%)
Cerebellum	1,219 (4.3%)	66 (1.5%)	142 (1.7%)
Brainstem	120 (0.4%)	5 (0.1%)	12 (0.1%)
Ventricle	96 (0.3%)	12 (0.3%)	20 (0.2%)
Overlapping	6,024 (21.3%)	606 (14.0%)	1,441 (17.7%)
Laterality			
Left	11,538 (40.8%)	1875 (43.4%)	3,139 (38.5%)
Mid	4,629 (16.4%)	275 (6.4%)	1,342 (16.4%)
Right	12,123 (42.9%)	2,168 (50.2%)	3,678 (45.1%)
Tumor extension			
Confined	21,523 (76.1%)	3,727 (86.3%)	6,664 (81.7%)
Ventricles	1,119 (4.0%)	132 (3.1%)	312 (3.8%)
Midline	4,493 (15.9%)	353 (8.2%)	948 (11.6%)
Metastasis			
Yes	398 (1.4%)	30 (0.7%)	73 (0.9%)
No	27,892 (98.6%)	4,288 (99.3%)	8,087 (99.1%)
BCSS			
Alive	4,686 (16.6%)	729 (16.9%)	1,288 (15.8%)
Dead	23,604 (83.4%)	3,589 (83.1%)	6,871 (84.2%)

GTR, gross total resection; SpTR, supratotal resection; BCSS, brain cancer-specific survival.

### Model performance

The C-index and IBS were calculated using the testing set to evaluate model discrimination. We trained the three-layered DeepSurv model, CPH model, and RSF on the overall training set and trained the BDE, BITES, DeepSurv, CPH model, and RSF on the GTR and SpTR training sets. The detailed model performance is presented in Table 2. For all patients, the CPH model exhibited the highest C-index (0.68; 95% CI, 0.67–0.69) and the lowest IBS (0.066; 95% CI, 0.062–0.071) (the lower the IBS, the better the performance). For the GTR group, BDE and the CPH model had the highest C-index (0.64; 95% CI, 0.61–0.66). However, the CPH model had a high IBS (0.104; 95% CI, 0.093–0.114). BITES had the lowest IBS (0.067; 95% CI, 0.060–0.077), followed by BDE (IBS, 0.068; 95% CI, 0.061–0.077). In the SpTR group, the CPH model had the highest C-index (0.68; 95% CI, 0.66–0.69), followed by BDE (0.67; 95% CI, 0.65–0.68). BDE had the lowest IBS (0.068; 95% CI, 0.062–0.077), followed by BITES (0.069; 95% CI, 0.063–0.078).

**Table 2:** Detailed model performance and treatment recommendation effects.

Model	Total		GTR		SpTR		DRMST^TaR^	HR^TaR^	DRMST^RST^	HR^RST^
	C-index	IBS	C-index	IBS	C-index	IBS				
BDE	–	–	0.64 (0.61–0.66)	0.068 (0.061–0.077)	0.67 (0.65–0.68)	0.068 (0.062–0.077)	5.90 (4.40–7.39) ^***^	0.71 (0.65–0.77) ^***^	2.54 (0.98–4.09) ^***^	0.86 (0.79–0.95) ^**^
BITES	–	–	0.63 (0.61–0.65)	0.067 (0.060–0.077)	0.66 (0.65–0.67)	0.069 (0.063–0.078)	4.95 (3.41–6.49) ^***^	0.75 (0.69–0.82) ^***^	4.12 (2.55–5.69) ^***^	0.78 (0.71–0.86) ^***^
DeepSurv	0.66 (0.65–0.67)	0.089 (0.084–0.097)	0.61 (0.57–0.62)	0.15 (0.14–0.17)	0.65 (0.65–0.68)	0.094 (0.086–0.107)	4.33 (2.78–5.88) ^***^	0.78 (0.71–0.85) ^***^	5.08 (3.55–6.61) ^***^	0.74 (0.68–0.81) ^***^
CPH	0.68 (0.67–0.69)	0.066 (0.062–0.071)	0.64 (0.61–0.66)	0.104 (0.093–0.114)	0.68 (0.66–0.69)	0.070 (0.063–0.077)	3.64 (2.07–5.21) ^***^	0.81 (0.74–0.89) ^***^	3.26 (1.69–4.83) ^***^	0.83 (0.75–0.91) ^***^
RSF	0.61 (0.60–0.62)	0.066 (0.062–0.071)	0.62 (0.60–0.65)	0.107 (0.096–0.118)	0.65 (0.64–0.66)	0.071 (0.063–0.079)	3.03 (1.51–4.56) ^***^	0.83 (0.76–0.91) ^***^	3.22 (1.54–4.89) ^***^	0.83 (0.76–0.92) ^***^

***p-value<0.001; **p-value<0.01; *p-value<0.05. DRMST, the difference of restricted mean survival time within 5 years between two groups; HR, hazards ratio calculated by the univariate Cox proportional hazard model. C-index, concordance index; IBS, integrated brier score. TaR, calculated individual treatment effect using survival time when the mortality rate is 50%; RST, calculated individual treatment effect using individual restricted mean survival time within 5 years. Total, model trained on overall patients; GTR, gross total resection group; SpTR, supra maximum total resection group. BDE, Balanced Decision Ensembles; BITES, Balanced Individual Treatment Effect for Survival data; CPH, Cox proportional hazards model; RSF, random survival forest.

To prevent the potential that the Consis. group may have better prognostic factors, the IPTW was used to correct the baseline imbalance between the Consis. and Inconsis. groups. Demographic and tumor characteristics were adjusted, including age, race, marriage status, income, report region, location, laterality, extension, tumor size, and metastasis status. Treatment variables were not adjusted as it was measured after exposure (treatment recommendation) and may introduce unmeasured confounding biases (29).

We calculated the dRMST and HR between the Rec. and Anti-rec. Groups based on the TaR and RST methods, respectively, as the core metrics to evaluate the model performance because they directly align with our core objectives of optimizing surgical treatment in patients with GBM. Table 2 shows the details of these metrics for each model, and the different ITE calculations are indicated using superscripts. BDE^TaR^ referred 485 (19.4%) patients for SpTR treatment; 1,008 (40.4%) patients’ actual treatments were consistent with the recommendation, and BDE^TaR^ achieved the highest dRMST (5.90; 95% CI, 4.40–7.39) and the lowest HR (0.71; 95% CI, 0.65–0.77). DeepSurv^RST^ (dRMST, 5.08; 95% CI, 3.55–6.61; HR, 0.74; 95% CI, 0.68–0.81) ranked second, which recommended 272 (10.9%) patients for SpTR, and its treatment consistency rate was 37.1%. BITES^TaR^ (dRMST, 4.95; 95% CI, 3.41–6.49; HR, 0.75; 95% CI, 0.69–0.82) ranked third, which recommended 179 (7.2%) patients for SpTR, and the Rec. group comprised 910 (36.5%) patients.

**Figure 2:**
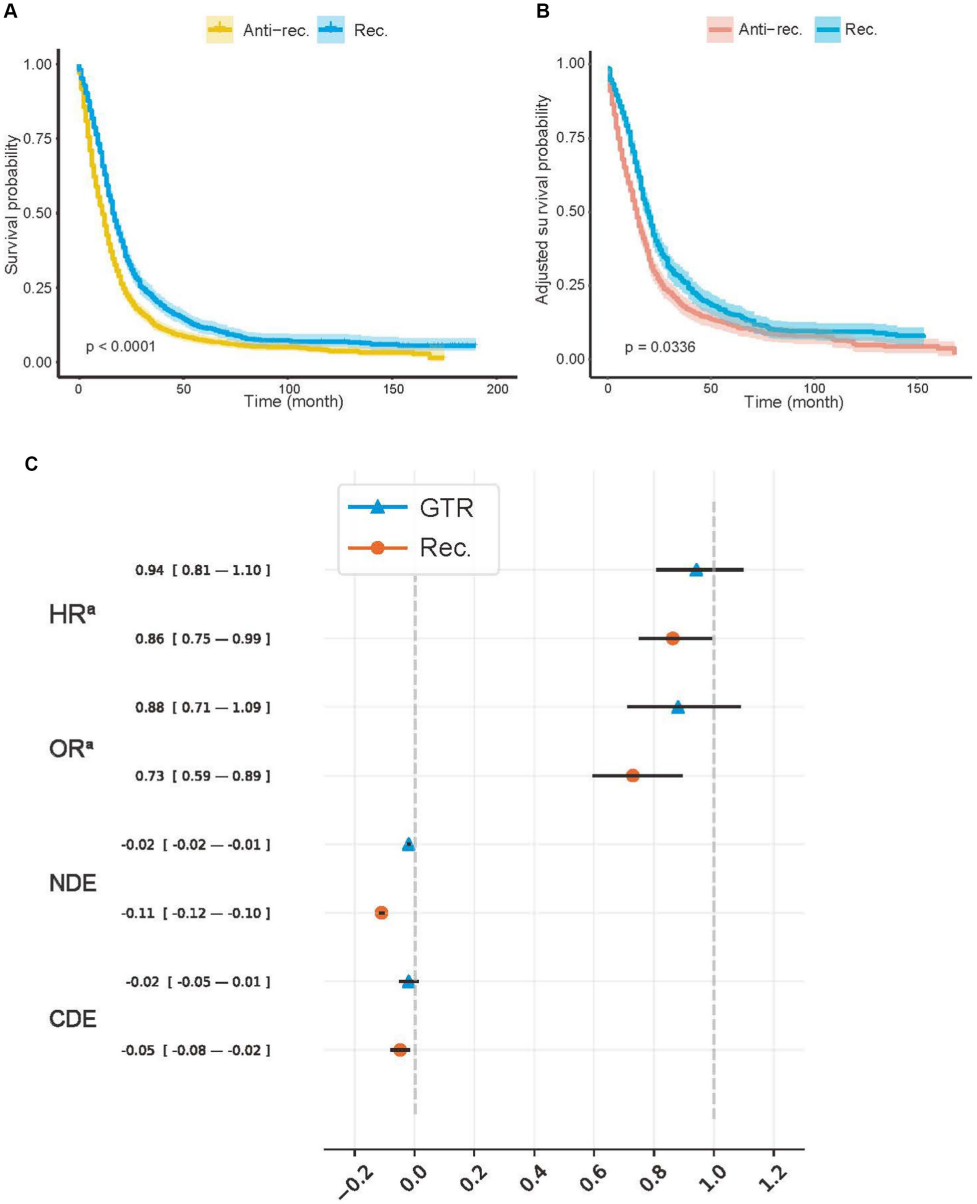
Average treatment effects of model recommendation and surgery. **(A)** Kaplan–Meier (K–M) curves of Anti-rec. vs. Rec.; **(B)** the inverse probability treatment weighting (IPTW)-adjusted K–M curves of Anti-rec. vs. Rec.; **(C)** average treatment effect (ATE) of model recommendation and surgery. Rec., patients’ actual treatment was consistent with the model recommendation; Anti-rec., patients’ actual treatment was inconsistent with the model recommendation; GTR, gross total resection; HR^a^, IPTW-adjusted hazard ratio; OR^a^, IPTW-adjusted odds ratio; NDE, natural direct effect; CDE, controlled direct effect. The IPTW was used to adjust preoperative baseline features between the tested groups. The *p*-value was calculated using a log-rank test with a two-sided significant threshold of 0.05. The NDE and CDE were calculated with treatment, including radiotherapy, chemotherapy, and surgery, as a mediator with a potential outcome framework.

In addition, we presented the detailed BCSS survival outcomes of the Rec. and Anti-rec. Groups of each method in Table 3, which included 5-year RMST, MST, and survival probability at 5 years (SaT) that was obtained from the life table. Based on the above results, the Rec. group of BDE^TaR^ had the best BCSS outcome (RMST [22.55; 95% CI, 21.35–23.74], MST [16; 95% CI, 16–18], SaT [11.63; 95% CI, 9.60–14.09]), and the Anti-rec. Group had the worst BCSS outcome (RMST [16.65; 95% CI, 15.76–17.55], MST [11; 95% CI, 10–12], SaT [7.32; 95% CI, 5.96–8.99]). We plotted the K–M curves of the Rec. and Anti-rec. Groups of BDE^TaR^ in Figure 2A and the inverse probability treatment weighting (IPTW)-adjusted K–M curves in Figure 2B, which make the K–M curves unbiased by covariates and treatment.

**Table 3:** Brain cancer-specific survival outcomes in each recommended group.

Model	Rec.			Anti-rec.			*p*-value
	RMST	MST	SaT	RMST	MST	SaT	
BDE^TaR^	22.55 (21.35–23.74)	16 (16–18)	11.63 (9.60–14.09)	16.65 (15.76–17.55)	11 (10–12)	7.32 (5.96–8.99)	<0.0001^***^
BDE^RST^	20.74 (19.46–22.01)	15 (14–16)	11.81 (7.78–12.45)	18.20 (17.32–19.09)	12 (12–13)	8.69 (7.29–10.36)	0.0020^**^
BITES^TaR^	22.18 (20.91–23.44)	16 (15–18)	11.90 (9.77–14.50)	17.22 (16.35–18.11)	12 (11–13)	7.41 (6.07–9.04)	<0.0001^***^
BITES^RST^	21.74 (20.44–23.05)	16 (14–17)	11.67 (9.49–14.36)	17.63 (16.76–18.50)	12 (11–13)	7.70 (6.37–9.32)	<0.0001^***^
DeepSurv^TaR^	21.84 (20.57–23.12)	16 (14–17)	11.21 (9.10–13.80)	17.51 (16.63–18.39)	12 (11–13)	7.88 (6.51–9.53)	<0.0001^***^
DeepSurv^RST^	22.22 (20.97–23.47)	16 (15–18)	12.11 (9.99–14.67)	17.15 (16.26–18.03)	12 (11–13)	7.24 (5.90–8.88)	<0.0001^***^
CPH^TaR^	21.47 (20.16–22.77)	16 (14–17)	11.10 (8.95–13.77)	17.83 (16.96–18.70)	12 (12–13)	8.05 (6.69–9.68)	<0.0001^***^
CPH^RST^	21.24 (19.34–22.54)	16 (14–17)	10.68 (8.56–13.33)	17.98 (17.11–18.86)	12 (12–13)	8.29 (6.92–9.94)	<0.0001^***^
RSF^TaR^	20.97 (19.74–22.21)	15 (14–16)	10.79 (8.76–13.29)	17.94 (17.04–18.84)	12 (12–13)	8.08 (6.68–9.76)	<0.0001^***^
RSF^RST^	21.39 (19.94–22.83)	15 (14–16)	10.82 (8.48–13.80)	18.17 (17.33–19.01)	13 (12–13)	8.40 (7.07–9.97)	0.0003^***^

^***^p-value<0.001, ^**^p-value<0.01,^*^p-value<0.05. Rec., patients’ treatment consistent with models’ recommendation; Anti-rec., patients’ treatment inconsistent with models’ recommendation. RMST, restricted mean survival time within 5 years; MST, median survival time (months); SaT, survival probability at 5 years (%); *p*-value, *p*-value of the log-rank test; TaR, calculated individual treatment effect using survival time when the mortality rate was 50%; RST, calculated individual treatment effect using individual restricted mean survival time within 5 years. BDE, Balanced Decision Ensembles; BITES, Balanced Individual Treatment Effect for Survival data; CPH, Cox proportional hazards; RSF, random survival forest.

We used IPTW-adjusted HR (HR^a^), IPTW-adjusted odds ratio (OR) (OR^a^), natural direct effect (NDE), and control direct effect (CDE) to measure the ATE of the Rec. group and the actual treatment (Figure 2C). We controlled all covariates for treatment and Rec. Additionally, the treatment was controlled for Rec. For CDE and NDE, treatment was viewed as a mediator to ensure that the protective effect or model recommendation was unbiased by treatment proportion. Both the GTR (−0.019; 95% CI, −0.025 to −0.014) and the Rec. group (−0.110; 95% CI, −0.119 to −0.101) showed a positive effect on survival according to the NDE values. The effect of the treatment group on survival time (HR^a^, 0.941; 95% CI, 0.807–1.098) and 5-year survival rate (OR^a^, 0.880; 95% CI, 0.711–1.089) (CDE: −0.019; 95% CI, −0.052 to 0.013) disappeared after controlling for confounding factors. However, the HR^a^ (0.862; 95% CI, 0.749–0.993), OR^a^ (0.729; 95% CI, 0.594–0.895), and CDE (−0.048; 95% CI, −0.079 to −0.016) values in the Rec. group suggested that model recommendations still showed significant protective effects on survival time and mortality.

### Model behavior and recommendation interface

**Figure 3:**
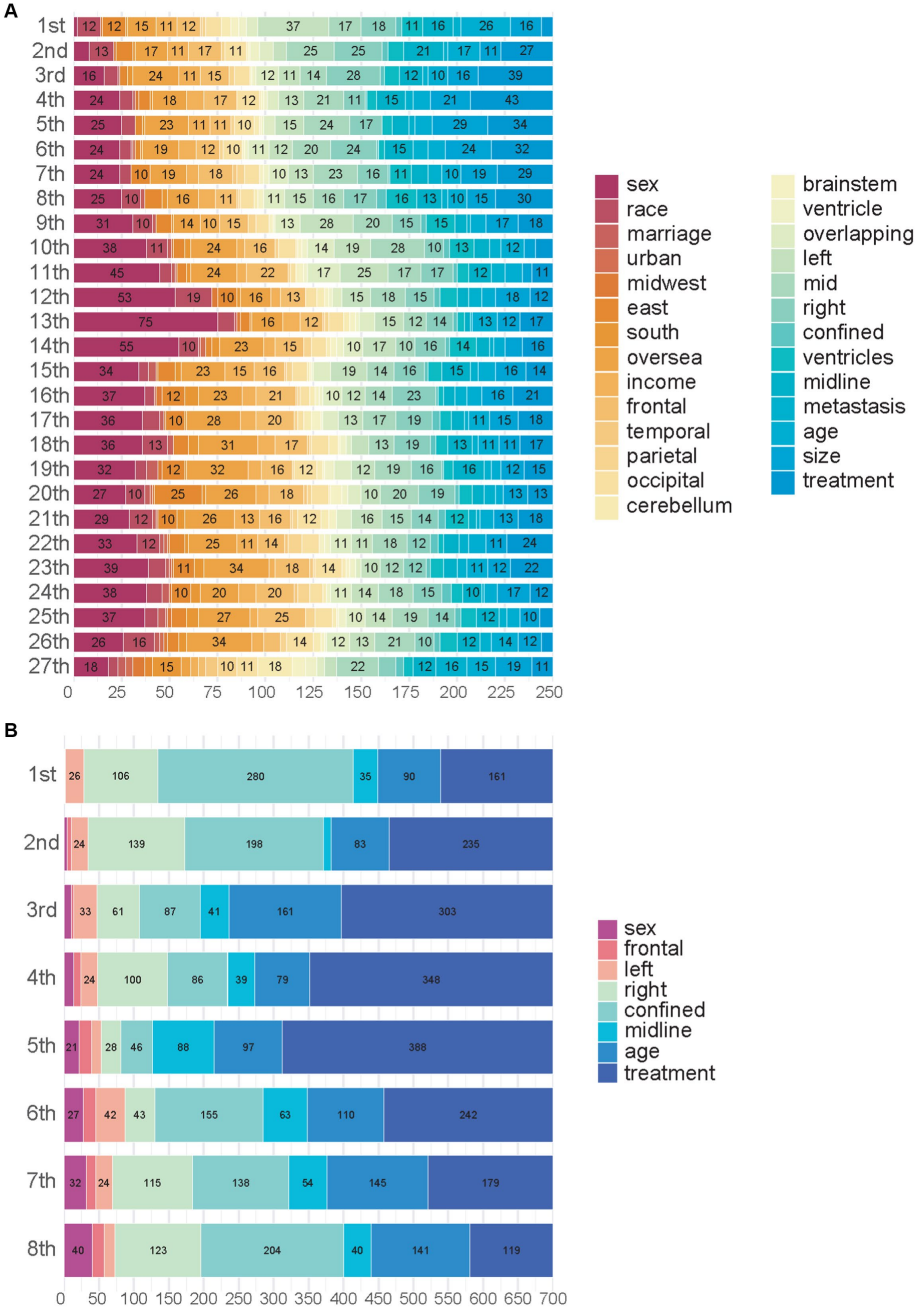
Importance of variables in Balanced Decision Ensembles. **(A)** Importance of variables in Balanced Decision Ensembles (BDE). **(B)** Top eight most important variables of BDE.

We used SurvSHAP(t), which is the first method introduced to date that can provide a time-dependent explanation with solid theoretical foundations, to explain the functional output of the models used in this study. Figure 3A shows the aggregation of variable rankings over 250 observations in the treatment recommendation testing set in the BDE, and Figure 3B visualizes the eight most important variables sorted by aggregated Shapley values over 700 observations in the same manner. The horizontal bars represent the number of observations for which the importance of the variable, represented as a given color, was ranked as first, second, and so on. Notably, treatment, including GTR and SpTR in BDE, was a sign of passing through different NODE and using different baseline hazards rather than a regular variable. In total, 280 (40.0%; 95% CI, 36.3–43.7%) observations indicated that confinement was the first important variable. Similarly, right laterality and age were considered the second and third critical variables, respectively, by the majority. This was followed by midline extension, left laterality, sex, and frontal tumor location.

**Figure 4:**
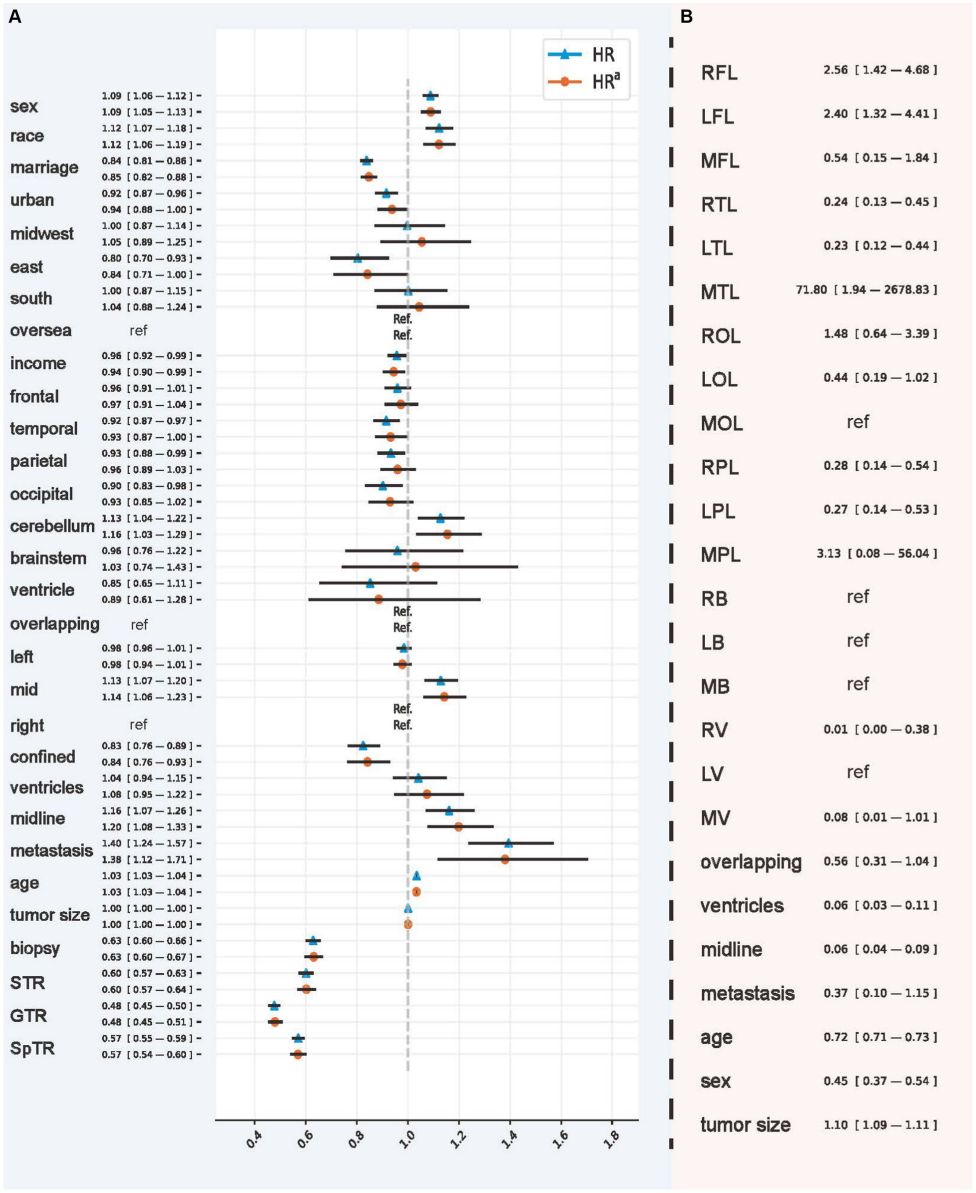
Hazards ratio of CPH and the odds ratio of model recommendation behavior. **(A)** The hazard ratio and inverse probability treatment weighting-adjusted hazard ratio obtained using the Cox proportional hazard model. **(B)** The odds ratio used to interpret the recommendation behavior of Balanced Decision Ensembles. RFL, right frontal lobe; LFL, left frontal lobe; MFL, middle frontal lobe; RTL, right temporal lobe; LTL, left temporal lobe; MTL, middle temporal lobe; ROL, right occipital lobe; LOL, left occipital lobe; MOL, middle occipital lobe; RPL, right parietal lobe; LPL, left parietal lobe; MPL, middle parietal lobe; RB, right brainstem; LB, left brainstem; MB, middle brainstem; RV, right ventricle; LV, left ventricle; MV, middle ventricle. The inverse probability treatment weighting was applied hierarchically based on the extent of resection.

In addition, we visualized CPH behavior using the HR values in Figure 4A, which had the best C-index and IBS in the testing set that included all patients. IPTW had a hierarchical correction for the EOR (HR^a^). According to the HR, in the overall population, patients were men (1.088; 95% CI, 1.058–1.120), were of advanced age (1.035; 95% CI, 1.033–1.036), and had tumors located in the cerebellum (1.128; 95% CI, 1.040–1.222) and the middle lobes of the brain (1.129; 95% CI, 1.065–1.196). Tumors with larger size (1.001; 95% CI, 1.0007–1.0013), crossing the midline (1.161; 95% CI, 1.069–1.261), and with metastases (1.395; 95% CI, 1.239–1.571) were unfavorable factors that significantly affected survival outcomes. In IPTW-adjusted values obtained controlling for confounding variables, the significance of the above variables remained. In contrast, HR values suggesting that tumors located in the temporal (0.915; 95% CI, 0.867–0.967), occipital (0.902; 95% CI, 0.832–0.978), and parietal (0.934; 95% CI, 0.881–0.989) lobes, confined *in situ* (0.825; 95% CI, 0.765–0.891), and undergoing biopsy (0.629; 95% CI, 0.601–0.658), STR (0.601; 95% CI, 0.573–0.631), GTR (0.477; 95% CI, 0.455–0.501), and SpTR (0.571; 95% CI, 0.547–0.595) were significantly protective of survival outcomes in patients with GBM. After IPTW adjustment, the significance of the above variables remained.

We used OR values to analyze the importance of demographic and tumor characteristics in the selection of GTR and SpTR (Figure 4B). The results showed that, compared with GTR, SpTR was more recommended for patients with GBM with tumors located in the right (2.562; 95% CI, 1.402–4.683) and left (2.398; 95% CI, 1.321–4.412) frontal and middle temporal lobes (71.803; 95% CI, 1.944–2678.834) and tumors with larger size (1.103; 95% CI, 1.094–1.111). However, compared with SpTR, GTR is a better choice for patients who are older (0.720; 95% CI, 0.708–0.733), who are men (0.446; 95% CI, 0.368–0.540), and whose tumors are located in the right (0.240; 95% CI, 0.129–0.447) and left (0.235; 95% CI, 0.125–0.443) temporal lobes, right (0.276; 95% CI, 0.141–0.539) and left (0.267; 95% CI, 0.135–0.530) parietal lobes, the right ventricle (0.011; 95% CI, 0.0003–0.380), multiple ventricles (0.056; 95% CI, 0.027–0.111), and across the midline (0.061; 95% CI, 0.042–0.086).

Supplementary Video S1 shows a prediction and treatment recommendation system that contains a CPH model and BDE. The system invokes the CPH model to predict the overall survival probability of a patient from the survival prediction view (right). In the treatment recommendation view (left), BDE was activated to predict the survival probability twice under the assumption that the patient underwent GTR or SpTR. ITE, indicating the BCSS benefits obtained by taking SpTR compared with GTR, calculated by the TaR and RST methods, enabled patients and physicians to make treatment choices with an intuitive and quantitative comparison of treatments. We also provided the mortality rate, RST, and TaR of the GTR and SpTR situation. The mortality of the actual situation was also presented. The user can select “Time” to obtain predicted values at different time horizons.

## Discussion

The prediction and explanation of ITE from censored time-to-event outcomes have received little attention in the data science domain (19, 30), which is surprising when one considers the enormous practical relevance of the subject (31, 32). The BITES framework uses strong ignorability (33) to remove confounding artifacts (34) and IPM to sufficiently balance the generating distributions of treatment groups on both latent representations (35, 36) and covariates (37). One key challenge in individualizing treatment recommendations is to reason about unbiased ITE (19, 22). Our results suggest that the combination of representation balancing strategy with T-learner can better control potential confounders and selection biases, as evidenced by the fact that BITES and BDE yielded a more significant protection effect compared to the traditional T-learners. We proposed BDE, a modified version of BITES, in which the treatment recommendation performance was further enhanced. This may be due to the better feature extraction ability of tree-based models, such as NODE, on structured data (38) and the feature selection ability of LassoNet. After thorough evaluations, adhering to the BDE recommendation can extend patients’ BCSS by 6 months within a span of 5 years, a benefit that clearly surpasses those who do not follow it.

In the treatment recommendation task, our core objective is to identify two subgroups that are heterogeneous for several treatments, thereby uncovering clinical features that can potentially guide the therapeutic intuitions of clinicians or can be directly applied to clinical practice. It was observed that, for the treatment recommendation problem, the C-index, although widely used, could not reflect the recommendation effect significantly well. For example, the CPH model and BDE had the same C-index in the GTR group, and the CPH model had a higher C-index than BDE in the SpTR group. However, the dRMST^TaR^ and HR^TaR^ of the CPH model were significantly lower than those of BDE. Taking the example of DeepSurv vs. the CPH model or RSF, IBS also did not fully respond to the recommendation effects, although the general trends were similar. Therefore, we propose using dRMST and HR as core evaluation metrics for the model, which directly reflect a better survival outcome in the treatment recommendation task. Another important reason is that dRMST and HR values have remarkably intuitive clinical significance (39, 40), are statistically guaranteed by well-established statistical methods (17), and can provide cross-sectional comparisons between models. The former measures the increase in the survival duration of patients during the follow-up period when adhering to the model’s recommendations compared to not following them, while the latter indicates the decrease in mortality during the same period. Consequently, these two metrics provide intuitive insights into the survival benefits of adhering to model recommendations from different angles.

Another phenomenon discovered was the significant effect of different ITE calculation methods on the recommendation effect. When using the same BDE model, the recommendation effect calculated by TaR is notably better than that calculated by the RST method, whereas RST showed a better result in DeepSurv. This further demonstrates the inappropriateness of using the C-index or IBS to evaluate the effectiveness of recommendations, as the same model is used in both ITE calculation processes. Similar trends were observed in other models, although the 95% CI showed no significant difference between the indicators. Our results indicate that, even when utilizing identical individual survival distributions, employing various methods for ITE calculation still significantly influences treatment recommendations. We observed that TaR is more applicable for GBM patients, probably because GBM patients usually have a shorter survival duration and the RST calculates the difference in survival over a certain period, which leads to a similar RST for all GBM patients, thereby making the ITE less sensitive. This warrants further investigation.

For clinical significance, based on the HR^a^, OR^a^, NDE, and CDE values obtained after correcting for confounders, treatment modalities consistent with the model recommendations were protective factors for patient survival, whereas neither GTR nor SpTR showed a significant effect, indicating that treatment recommendations using the model are more beneficial for prolonging the survival of patients with GBM.

In the total population of this study, based on IPTW-corrected HR values, we found that the important variables affecting the predicted survival outcomes of CPH were demographically related to age, sex, marriage, income, and urban area and tumor-related variables, including tumor location in the temporal lobe and cerebellum, laterality as intermediate, confinement *in situ*, crossing the midline, and tumor metastasis. Using SHAP values in patients undergoing GTR and SpTR, in addition to trends similar to those described above for the total population, we found that the location of the tumor in the frontal lobe and its left lateralization and right lateralization were also key variables affecting survival outcomes. Most of the variables derived to influence the prediction of survival outcomes were consistent with important prognostic factors for patients with GBM in previous studies (1, 41, 42), indicating that the model predictions can be supported by clinical research evidence.

Subgroup analyses were made through OR values, which showed a clear tendency for GTR to be more recommended for elderly (43) and male patients and for patients with GBM whose tumors were located in the right and left temporal and parietal lobes, the right ventricle, multiple ventricles, and across the midline, whereas SpTR was recommended for patients whose tumors were located in the right and left frontal and middle temporal lobes and those with larger tumor size. Most previous studies have focused on the effect of different EOR on survival time (3, 44, 45), with fewer findings on how to select resection scopes in different populations and patients with different tumor characteristics at the same time. Among the important characteristics on the basis of which the model recommended different EOR, age (46), sex (47), tumor size (48), and crossing the midline (49) were considered to interact with EOR in the prognosis of patients with GBM in previous studies; that is, the effect of EOR on survival outcome was specific to the above variables. As for the tumor location and laterality, the finding that patients with tumors located in the right frontal lobe are more suitable for SpTR is consistent with the recent expert consensus (50). However, our conclusions quantified the impact of these baseline characteristics on EOR selection and used multivariate regression to control for the cofounders. Thus, these findings help to provide individualized statistical evidence for clinical practice and deserve to be further validated in subsequent studies.

However, according to the HR, HR^a^, NDE, and CDE values, we found that SpTR was a risk factor in the overall trend for patients with GBM compared with GTR. This is inconsistent with the conclusion of most previous studies that SpTR prolongs survival compared with GTR (51, 52), which may be related to the insufficient sample size of previous studies owing to the aggressive nature of GBM and the limitations of clinical and methodological heterogeneity of RCT studies, demonstrating the superiority of this study in solving the controversial choice of treatment. Therefore, our study shows that ML models can use big data to analyze findings that are difficult to derive from RCT experiments. Different from traditional methods, the model can predict survival and make personalized recommendations, reducing unnecessary treatment risks and improving patient benefits. While the results will require additional experimental validation in the future, they are promising for guiding clinicians through the decision-making process to generate a new and comprehensive clinical prognostic analysis for GBM surgery.

To facilitate discussion of different potential surgical options, clinicians and patients need an informative tool that focuses on survival benefits. In real cases, the establishment of a graphic treatment recommendation system (Supplementary Video S1) with multiple individual survival and comparison indicators will be key in effectively conveying results and illustrating complex analyses to patients, family members, and doctors. Treatment recommendation and survival prediction results from models create a visualized and quantified platform that allows patients to directly compare the survival advantages between different therapies and choose the optimal treatment plan based on their preferences.

## Limitations

Due to SEER database limitations, there was a lack of some key information in the study, such as IDH mutation and Karnofsky Performance Status Scale score. However, this study confirms the feasibility of DL models to provide treatment recommendations for patients with GBM. Further studies are advocated to include more clinically advanced features to achieve even more accurate prediction and implement more advanced DL models and the TaR method that calculates ITE.

## Conclusion

This study is the first to use the DL approach that combines important variables pertaining to demographics and oncology for survival analysis, treatment recommendations, and visual presentation for GBM patients. The potential of BDE to assist in clinical treatment decision-making is evident, as clearly evidenced by its superior efficacy in treatment recommendations. The model identifies patients with tumors in the right and left frontal and middle temporal lobes, as well as those with larger tumor sizes, as optimal candidates for SpTR.

## Data availability statement

The original contributions presented in the study are included in the article/Supplementary material, further inquiries can be directed to the corresponding authors.

## Ethics statement

Ethical approval was not required for the studies involving humans because this study analyzed public datasets which can be found at: the Surveillance, Epidemiology, and End Results Program (https://seer.cancer.gov/index.html). The studies involving human participants were approved by the national cancer institution. Written informed consent for participation was not required for this study in accordance with the national legislation and the institutional requirements. The studies were conducted in accordance with the local legislation and institutional requirements. The participants provided their written informed consent to participate in this study.

## Author contributions

EZ: Conceptualization, Data curation, Investigation, Methodology, Software, Writing – original draft. JW: Data curation, Investigation, Methodology, Writing – original draft. QJ: Conceptualization, Data curation, Formal analysis, Investigation, Writing – original draft. WS: Data curation, Investigation, Methodology, Writing – original draft. ZX: Conceptualization, Formal analysis, Investigation, Methodology, Writing – original draft. PA: Data curation, Formal analysis, Investigation, Methodology, Writing – original draft. ZC: Conceptualization, Data curation, Formal analysis, Methodology, Writing – original draft. ZD: Data curation, Formal analysis, Writing – original draft. DS: Project administration, Resources, Supervision, Writing – review & editing. ZA: Funding acquisition, Project administration, Supervision, Writing – review & editing.
